# Upregulation of exosomal circPLK1 promotes the development of non-small cell lung cancer through the miR-1294/ high mobility group protein A1 axis

**DOI:** 10.1080/21655979.2022.2026727

**Published:** 2022-02-03

**Authors:** Chuankui Li, Guowen Wang, Xiaoxiao Ma, Tao Tao, Qicai Li, Yifan Yang, Haiwei Sang, Zuyi Wang

**Affiliations:** Department of Thoracic Surgery, The First Affiliated Hospital of Bengbu Medical College, Bengbu, China

**Keywords:** NSCLC, circPLK1, miR-1294, HMGA1

## Abstract

CircRNAs (circular RNAs) have been implicated in the development and progression of a variety of cancers. The molecular pathways underlying the progression of NSCLC (Non-Small Cell Lung Cancer) and the associated regulation of circRNAs in NSCLC remain to be fully elucidated. In this study, we found that circPLK1 expression was upregulated in serum exosomes and tissues from NSCLC patients. The Kaplan–Meier survival analysis revealed that a high expression level of circPLK1 was associated with a poorer prognosis in NSCLC patients. Exosomes extracted from NSCLC serum could promote the replication, migration, and invasion of NSCLC cells and suppress apoptotic cell death. The overexpression of circPLK1 also promotes the malignant phenotype of NSCLC cells. Molecular analyses demonstrated that circPLK1 directly targets miR-1294 and negatively regulates its activity. And circPLK1 overexpression facilitates the progression of NSCLC by negatively regulating miR-1294 level and maintaining a high-level expression of HMGA1 (High Mobility Group Protein A1). Our study suggests that circPLK1 upregulation plays an important role in NSCLC progression by targeting miR-1294/HMGA1 axis. These data provide a theoretical basis for the development of therapeutic strategy targeting exosomal circPLK1 in NSCLC treatment.

## Introduction

Lung cancer is one of the most prevalent cancers and the leading cause of cancer-related mortality worldwide [1]. NSCLC (Non-Small Cell Lung Cancer) accounts for nearly 85% of lung cancer diagnosis [2]. Despite the advancements in novel anticancer therapies such as targeted therapies and immunotherapies, the prognosis for lung cancer patients still remains low. Five-year survival rates decline precipitously from 59% in locally advanced tumors to 32% in regionally advanced tumors, with only 6% for metastatic tumors [1]. The high mortality rate associated with NSCLC is primarily a result of the late-stage diagnosis in many cases, where the therapy is less likely to be effective [2].

Recent advances in next-generation sequencing technology and bioinformatics have revolutionized our understanding of RNA biology in cancer, especially with the identification of noncoding RNAs (ncRNAs) regulating diverse biological processes [3]. CircRNA (Circular RNAs) are closed-loop non-coding RNAs which could bind to target microRNAs, acting as a ‘sponge’ to competitively prevent the biding of miRNA to the downstream target mRNA [4]. Recent studies revealed that the abnormal expression of circRNAs could regulate tumor progression and metastasis [5,6]. For instance, circRNA ciRS-7 expression was upregulated in colorectal carcinoma, which was correlated with a poorer patient survival [7,8]. In contrast, in gastric cancer, the increased circPVT1 expression level was an independent predictor of improved overall survival and disease-free survival [9]. CircRNAs usually target miRNAs to regulate different aspects of tumor biology. For instance, Lin et al. demonstrated that in breast cancer the downregulation of circPLK1 suppresses tumor growth by increasing miR-4500 level and decreasing the expression of IGF1 (Insulin Like Growth Factor 1) [10].

Exosomes are secreted membrane-encapsulated vesicles containing proteins, lipids, DNA, and RNA originating from the host cell. The exosomes secreted by tumor cells could modulate cellular functions in tumor microenvironment and facilitate tumor proliferation and invasion [11–13]. Additionally, exosomes also contribute to the establishment of metastatic colonization [13]. Interestingly, tumor-associated exosomes also contain non-coding RNAs such as circRNAs and miRNAs, which are believed to modulate the tumor development [11–13].

The current research focused on a newly emerging circRNA (circPLK1) in tumor biology and investigated its expression and role in NSCLC. Its expression was analyzed in clinical NSCLC samples and cell lines. The StarBase database was used to predict the interactions among circRNA-miRNA-mRNA. We identified and validated the function involvement of CircPLK1/miR-1294/HMGA1 axis in promoting NSCLC progression. Our study provides a theoretical basis for the development of therapeutic strategy targeting circPLK1 in NSCLC treatment.

## Materials and methods

### Cell culture and transfection experiment

CALU3, CALU6, A549, H1229, and H1975 cells were obtained from the American Type Culture Collection (ATCC, Manassas, VA) and cultured at 37°C in DMEM medium supplemented with 10% fetal bovine serum (FBS) and 1% penicillin-streptomycin solution (Gibco, USA). Human bronchial epithelial cell line (HBE1) extracted from airway tissues was cultured in Clonetics BEGM medium with hormone additive (Cambrex Lonza, East Rutherford, NJ).

Stable overexpression of circPLK1 and the downregulation of HMGA1 were established by lentiviral transduction. pLKO.1-Puro lentiviral vector was used for the cloning circPLK1 or HMGA1 shRNA sequencing. Lentiviral plasmids overexpressing circPLK1 or carrying HMGA1 shRNA and sh-Negative Control (sh-NC) were constructed by GenePharma Co. Ltd. (Shanghai, China). The packaging of recombinant lentivirus was performed in 293 T cells by GenePharma Co. Ltd. (Shanghai, China). To generate stable cell lines, 1 × 10^5^ cells were seeded in a 24-well plate. When cells reached at 50 ~ 60% confluence, cells were infected with recombinant lentivirus at a MOI (multiplicity of infection) = 5, in the presence of 10 µg polybrene (Sigma, tr-1003-g). Infected cells were selected with 1.0 μg/mL puromycin for 2 weeks to eliminate the uninfected cells before further experiment. qPCR and Western blot were performed to confirm the efficiency of overexpression and knockdown.

miR-1294 mimic, miR-NC, siRNA targeting circPLK1 and control siRNA were synthesized by GenePharma Co. Ltd. (Shanghai, China). Cell transfection was performed using Lipofectamine® 3000 reagent (Thermo Fisher Scientific, L3000001). In 6 well plate, 60% confluent cells were transfected with 100 nM of microRNA mimic or siRNA according to manufacturer’s instruction. Transfected cells were subjected to subsequent analysis 48 hours post-transfection.

### Sample collection

Between May 2020 and June 2021, 50 paired NSCLC tumors and their adjacent normal tissues were collected from NSCLC patients at the first affiliated hospital of Bengbu medical college. The patients undergone video-assisted thoracoscopic surgery (VATS) wedge resection of lung between Lobectomy, which was authorized by Bengbu Medical College Ethics Committee (No:2020–193). Before surgery, patients had not received chemotherapy or radiotherapy. Collected tissue specimens were snap-frozen in liquid nitrogen and then stored at −80°C deep freezer. The clinicopathological characteristics of the patients are summarized in Table 1. All the patients signed an informed consent before being enrolled in the study.

**Table 1.** Correlations of CircPLK1 expression with clinicopathologic features of NSCLC patients

Variable	Number	CircPLK1 expression		*P*-value
		Low	High	
Age (years)				0.382
<60	19	11	8	
≥60	31	14	17	
Gender				0.564
Male	30	14	16	
Female	20	11	9	
Tumor size (cm)				0.569
<3	22	12	10	
≥3	28	13	15	
TNM				0.031
I–II	21	17	4	
III–IV	29	8	21	
Distant metastasis				0.025
No	19	16	3	
Yes	31	9	22	
differentiation				0.529
High	14	8	6	
Low	36	17	19	

For exosome purification, 4 ml of blood was obtained from NSCLC patients for. Additionally, blood samples from healthy volunteers were taken as a control group. The blood samples were centrifuged at 2300 rpm for 15 minutes to isolate the serum. The serums samples were further centrifuged at 12,000 g for 15 minutes to remove residual cells and debris. The supernatant was collected and deposited at −80°C for long-term storage for further exosome isolation.

### Purification of exosomes from serum

The procedure of exosome purification was adapted from a previous study [14]. Briefly, the thawed serum was combined with PBS at a ratio of 1:1. The mixture was centrifuged at 2000 g for 30 minutes at room temperature (RT) and 12,000 g for 45 minutes at 4°C. Then the supernatant was further centrifuged at 4°C at 110,000 g for 2 hours. The precipitate was resuspended in PBS and centrifuged at 110,000 g for 60 minutes at 4°C. The resulted exosome pellet was stored at −80°C until required.

### Exosome characterization and quantification

Exosomes were resuspended in nuclease-free water, and a fraction of the solution was placed on a carbon-coated copper mesh and mixed with 2% uranyl acetate. After drying, the samples were analyzed under an electron microscope JOEL JEM-1200 EX II (INTA Castelar, Argentina).

Purified exosomes were dissolved in PBS before being analyzed using the Nanoparticle Tracking Analysis method (NTA). The size distribution and concentration of diluted exosomes were determined using nanoparticle tracking analysis (NTA) on a Nanosight NS300TM (Malvern Instruments, UK).

### Exosome uptake assay

A549 and H1299 cells were irradiated with 8 Gy, and isolated exosomes from the serum of NSCLC patients were applied to the cell culture medium. Cells were washed twice with PBS after 16-hour incubation. Cells were harvested and resuspended in PBS, and their fluorescence intensity was determined using a fluorescence cell analyzer.

### Purification of total RNA and quantitative reverse transcription-polymerase chain reaction (qRT-PCR)

Total RNA was purified according to the manufacturer’s instructions using the RNeasy Mini Kit (QIAGEN, Hilden, Germany). 1 μg of total RNA was used for reverse-transcription into cDNA using a One-step PrimeScript cDNA synthesis Kit (Takara Bio Inc.). The qPCR was conducted using SYBR Premix Ex Taq (Takara Bio Inc.) on a 7500 Quick Dx Real-Time PCR instrument (Applied Biosystems, Waltham, MA, USA). The cycling conditions are as follows: 10 min at 50°C and 3 min at 95°C, 45 cycles of 95°C 5 sec, 55°C 30 sec, and 72°C 30 sec. Finally, the 2–∆∆Ct method was used to analyze the relative expression level and GAPDH was used as the internal reference gene. All primer sequences were synthesized and purchased from Shanghai Sangon Biotechnology Co., Ltd. (Shanghai, China).

### Western blot analysis

Cells in the logarithmic growth phase were lysed for 30 minutes with RIPA lysis buffer, and then centrifuged at 12,000 g for 5 mins. Protein quantification was performed using the Micro BCA Protein Assay Kit (Pierce, Rockford, IL). 10 ug protein was used for SDS-PAGE electrophoresis. Separated protein in SDS-PAGE gel was transferred onto the PVDF membrane (BioRad 1620177, Irvine, CA, USA). After blocking with 5% skimmed milk for 1 hour, the membrane was then incubated with primary antibodies: rabbit mAb CD63 (1:1,000; Abcam), rabbit mAb Hsp70 (1:1,000; StressGen), rabbit mAb HMGA1 (1:100; Abcam), and rabbit pAb -actin (1:1,000, Cell Signaling Technology). The primary antibodies were dissolved in TBST comprising 5% BSA and incubated overnight at 4°C. The membrane was washed 3 times with TBST for 5 minutes each. After wash, the membrane was further incubated with goat anti-rabbit secondary antibody conjugated with HRP (1:3000; Cell signaling #7074, MA, USA). Then the membrane was washed 4 times with TBST and the protein bands were visualized using an EZ-ECL Chemiluminescence Detection kit (Pierce, Rockford, IL) and photographed on a gel imager system (Bio-Rad, Hercules, CA, United States). The densitometry analysis was performed with Image J software (Bethesda, MD, USA).

### Assay for cell viability

Cells were seeded in to a 96-well plate at a density of 1500 cell/well and cultured in a humidified cell culture incubator for 0, 24, 48, 72 and 96 hours, respectively. Subsequently, 10 μL CCK8 reaction solution (Solarbio, CA1210, Beijing, China) was added to the cell culture at indicated time point and incubated for 1 hour in a humidified cell culture incubator. The light absorption value (OD value) in each condition was captured at 450 nm wavelength on a Synergy H1 microplate reader (Winooski, Vermont, USA).

### Analyses of apoptosis

The detection of cell apoptosis was performed using the FITC Annexin V Apoptosis Detection Kit (BD Biosciences, PharMingen, San Jose, CA, USA) according to the manufacturer’s instructions. Cells with different treatments were trypsinized and washed twice with PBS, and resuspended in the staining solution. In brief, 5 μL Annexin V-FITC and 5 μL PI were added to the 1000 μL cell resuspension with 1 million cells and incubated for 30 mins in the dark. Stained cells were centrifuged and washed twice with 1PBS and resuspended in 400 μL PBS. The percentage of apoptotic cells was detected by BD FACS CantoTM II Flow Cytometer (BD Biosciences).

### Transwell invasion assay

The transwell upper chamber (Corning, NY, USA, #3401) was used for invasion assay. The upper chamber surface was coated with 50 mg/L Matrigel at 1:8 dilution in cold PBS, and then filled with the diluted polycarbonate film, while the transwell upper chamber without Matrigel was used for migration assay. 5 × 10^5^ cells were inoculated into the transwell upper chamber in serum-free medium and 2 mL of 10% serum-containing medium was added to the lower chamber. After incubation (24 hours for migration assays; 48 hours for invasion assays), culture medium was discarded and the cells were fixed with 4% paraformaldehyde at room temperature for 10 mins and stained with 0.5% crystal violet (Sigma, Germany, #109218) for 20 mins. Cells were photographed under Leica AM6000 microscope ((Leica, Wetzlar, Germany). Cells were counted and evaluated in five random fields of view.

### Assay for colony-forming

Cell with indicated treatment were trypsinized and resuspended in culture medium. Cells were seeded into a 6-well plate (1000 cells/well) and cultured for 14 days, and the culture medium was changed every 3 days during the period. After 14 days, cells were fixed with 4% paraformaldehyde at room temperature for 10 mins and stained with Giemsa reagent (Giemsa Stain Kit, Abcam ab150670) or 0.5% crystal violet (Beyotime, Shanghai, China) for 20 mins. Subsequently, the number of colonies was counted and the morphology of the colonies was photographed under Leica AM6000 microscope (Leica, Wetzlar, Germany).

### Dual-luciferase reporter assay

To demonstrate the functional interaction between circPLK1/miR-1249, and miR-1294/HMGA1, the sequence containing the wild-type binding site and the sequence with mutated binding site were cloned into the PmirGLO vector (Promega, E1330). The reporter plasmid and Renilla luciferase (hRlucneo) control plasmid were co-transfected into cells with either miR-1249 mimic or miR-NC in a 12-well plate (1 × 10^5 cells/well) using Lipofectamine 3000 reagent according to the manufacturer’s instructions (Invitrogen, L3000001). 48 h post transfection, the relative luciferase activities were measured using PierceTM Renilla-Firefly Luciferase Dual Assay Kit (Thermo Fischer Scientific, USA) on a luminescence microplate reader (Infinite 200 PRO; Tecan). The relative firefly luciferase activity in the reporter plasmid was normalized to that of Renilla luciferase (hRlucneo) control plasmid.

### RNA pull-down assay

Biotinylated miR-1294 and biotinylated miR-NC were transfected into A549 and H1299 cells, respectively. 48 hours post transfection, cells lysates were collected by IP lysis buffer (Beyotime, P0013) and incubated with 100 µL M-280 streptavidin magnetic beads (Sigma-Aldrich, 11205D) at 4°C shaking overnight. 10% of the lysates was saved as the input. A magnetic bar was used to pull down the magnetic beads and associated nucleic acids, and then the samples were washed 4 times with high salt wash buffer. Purification of the RNA from the input and the elutes of the pull-down samples was performed using the TRIzol® reagent (Invitrogen, Carlsbad, CA, USA), and the RNA was quantified by qRT-PCR analysis.

### RNA immunoprecipitation (RIP) assay

Cells were lysed using IP lysis buffer (Beyotime, P0013) and incubated with Pierce™ Protein A/G Magnetic Beads (Thermo Fisher Scientific, 88803) conjugated with a rabbit anti-Ago2 (Abcam, ab32381) antibody or with a negative control normal rabbit anti-IgG (Abcam, ab188776). The mixture was incubated at 4°C with shaking overnight. Magnetic beads were precipitated using a magnetic bar and the precipitated samples were washed three times with lysis buffer. The eluted samples were purified using the TRIzol® reagent (Invitrogen, Carlsbad, CA, USA), and the RNA was quantified by qRT-PCR analysis.

### Statistical analysis

Statistical analyses were performed with SPSS 20.0 software (IBM SPSS, Armonk, NY, USA). All the experiments were repeated at least three times. The association between circPLK1 expression and the clinic pathological parameters was evaluated with Chi-square analysis. The statistical difference between two groups was compared using unpaired student’s t tests. Comparisons among multiple groups were analyzed using one-way analysis of variance (ANOVA) with Tukey’s post hoc test for pairwise comparison. Comparisons of data at multiple time points were examined using two-way ANOVA. Kaplan Meier Curve and log-rank test were used to compare the cumulative survival rates in NSCLC patients. Spearman correlation analysis was performed to determine the correlation between expression levels. The region under the receiver operating characteristic (ROC) curve (AUC) was calculated to determine the specificity and predictability of circPLK1. Data were reported as the mean ± standard deviation (SD). P < 0.05 was considered to be statistically different.

## Results

In this study, we showed that circPLK1 was upregulated in serum exosomes from NSCLC patients, and a high expression level of circPLK1 was associated with a poorer prognosis in NSCLC patients. Exosomes extracted from NSCLC serum promote the replication, migration, and invasion of NSCLC cells and suppress apoptotic cell death. CircPLK1 overexpression also promotes the malignant phenotype of NSCLC cells. Molecular analyses demonstrated that circPLK1 directly targets miR-1294 and negatively regulates its activity. CircPLK1 overexpression facilitates the progression of NSCLC by negatively regulating miR-1294 level and maintaining a high-level expression of HMGA1 (High Mobility Group Protein A1).

### Samples

**Figure 1.**
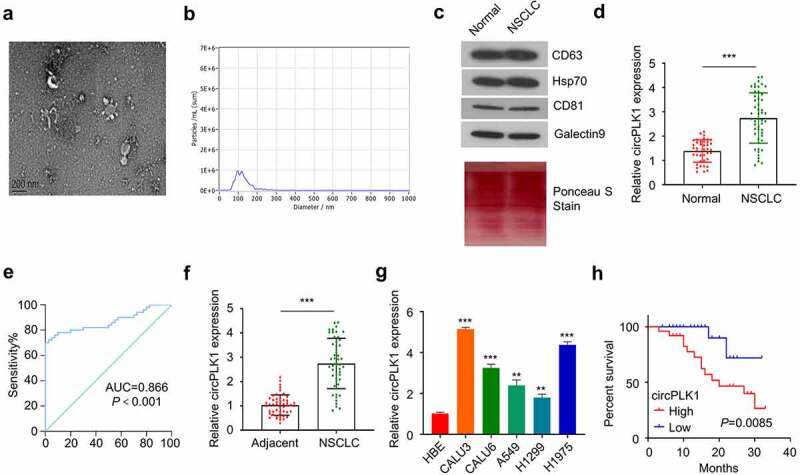
Identification of circPLK1 in NSCLC and its prognostic significance. (a) The exosomes derived from the serum of NSCLC patients and healthy subjects were analyzed using TEM. (b) The size of exosomes was evaluated by NTA analysis. (c) HSP70, CD63, CD81 and Galectin9 were detected using Western blotting in exosome samples.Total protein staining was shown as the loading control. (d) CircPLK1 expression in serum exosomes of NSCLC patients and healthy controls was quantified by qRT-PCR. (e) ROC curve was used to analyze the circPLK1 predictability. (f) CircPLK1 expression in NSCLC tissues and adjacent normal tissues. (g) The relative expression level of circPLK1 in a panel of NSCLC cell lines. (h) Kaplan Meier Curve and log-rank test were used to compare the overall survival in NSCLC patients. *P < 0.05; **P < 0.01; ***P < 0.001.

The present study enrolled 50 patients with NSCLC, and their clinical characteristics are summarized in Table 1. TEM was used to characterize exosomes derived from the serum of NSCLC patients. Purified exosomes showed a vesicular morphology (Fig. A). NTA analysis of the purified exosome samples showed a standardized community of nanoparticle size (50–200 nm) (Figure 1(b)). Finally, Western blotting analysis was performed to validate the presence of exosomal-specific markers (HSP70, CD63, CD81 and Galectin9) in the purified exosome samples (Figure 1(c)).

### CircPLK1 is upregulated in NSCLC tumor samples and cells

We obtained 50 blood samples from NSCLC patients and 40 samples from healthy controls to determine the extent of circPLK1 expression in serum exosomes. circPLK1 expression was significantly higher in the exosomes from NSCLC patients than that of the healthy donors (Figure 1(d)). The receiver operating characteristic curve was used to assess the predictability of exosome circPLK1 in NSCLC. As shown in Figure 1(e), the region under the receiver operating characteristic curve (AUC) was more 0.866, which shows significant value for predicting NSCLC. CircPLK1 expression was also significantly upregulated in NSCLC tissues as compared to the adjacent normal tissues (Figure 1(f)). Following that, we examined the expression of circPLK1 in a panel of NSCLC cell lines (CALU3, CALU6, A549, H1229, and H1975) and a human bronchial epithelial cell line (HBE). RT-qPCR analysis revealed that NSCLC cells expressed a significantly higher level of circPLK1 than HBE cells (Figure 1(g)). A549 and H1229 cells which express a relatively lower level of circPLK1 were selected for the following study to investigate whether circPLK1 overexpression could promote the malignant phenotype of the NSCLC cells.

To examine whether circPLK1 expression level correlates with the survival of NSCLC patients, the patients were divided into high expression and low expression groups based on the median expression level of circPLK1 in NSCLC tissues. A high circPLK1 expression level was correlated with a poor prognosis in NSCLC patients (Figure 1(h)). Moreover, high circPLK1 expression was also associated with advanced TNM staging (size) and distant metastasis in NSCLC patients (p < 0.05) (Table 1). Our findings indicate that the upregulation of circPLK1 favors the profession of NSCLC, which may serve as a predictor of poor prognosis in patients with NSCLC.

### NSCLC-derived serum exosomes promote cell growth and metastatic properties in NSCLC cells

**Figure 2.**
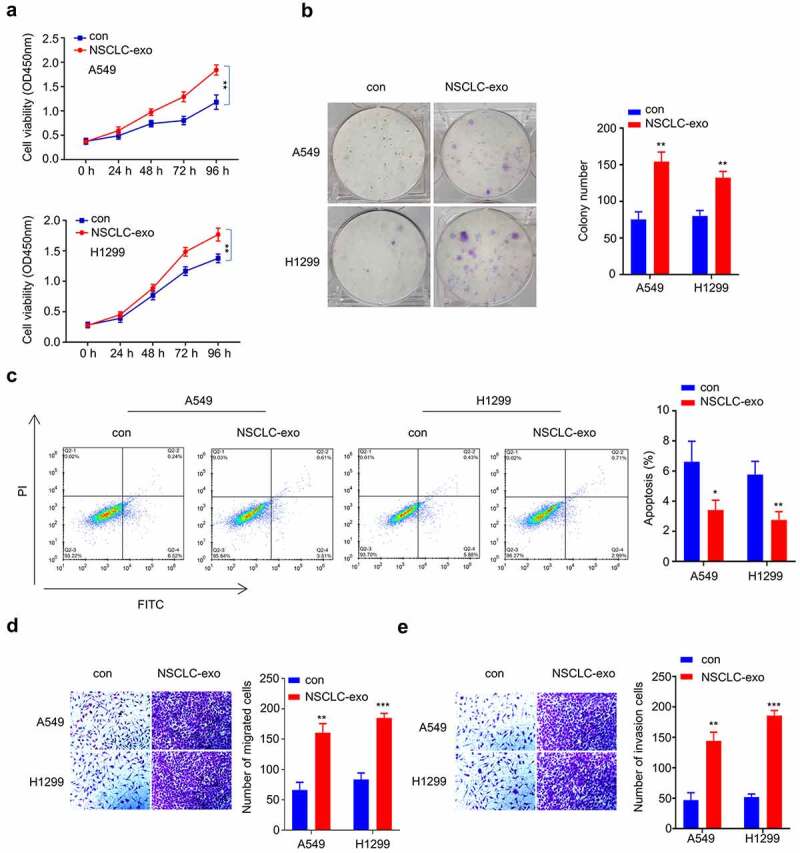
Serum exosomes from NSCLC patients promote the proliferation, migration and invasion of NSCLC cells. (a) CCK-8 assay was used to detect cell proliferation. (b) Colony formation assay was used to examine the ability of colony formation. (c) Apoptosis was detected by flow cytometry. (d) Transwell experiment was performed to examine the ability of different groups to migrate. (e) Transwell assay was used to determine the invasive ability of different groups. *P < 0.05; **P < 0.01; ***P < 0.001.

**Figure 3.**
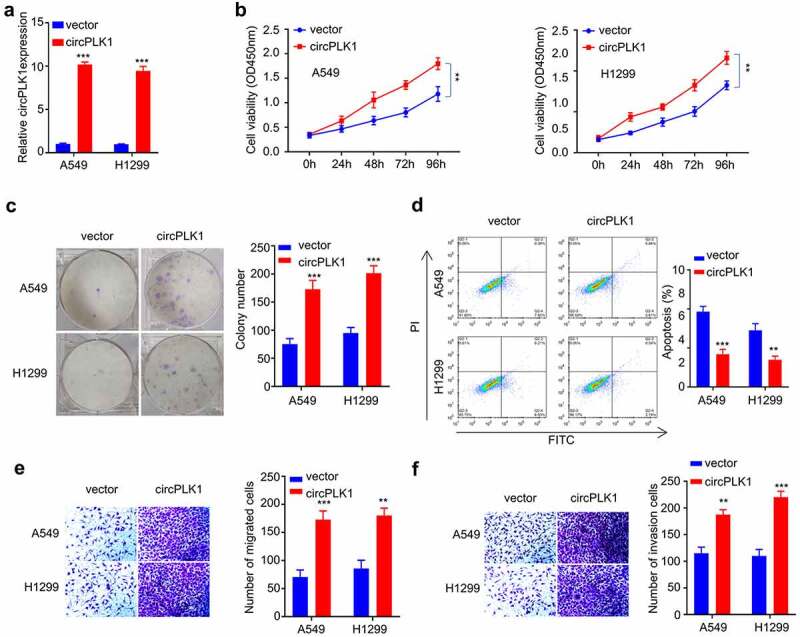
Overexpression of circPLK1 promotes the proliferation, migration, invasion in NSCLC. (a) Overexpression of CircPLK1 was detected by qRT-PCR. (b) CCK-8 assay was used to detect cell proliferation. (c) Colony formation assay was used to examine the ability of colony formation. (d) Apoptosis was detected by flow cytometry. (e) Transwell assay to measure migration ability. (f) Transwell assay to measure invasion ability. *P < 0.05; **P < 0.01; ***P < 0.001.

To determine the functional role of the exosomes derived from NSCLC serum, A549 and H1229 cells were cultured with exosomes from NSCLC samples (NSCLC-Exo) or healthy control (con) for 24 h, respectively. We then performed functional assays to determine whether exosomes derived from NSCLC serum promote the malignant phenotype in NSCLC cells. A549 and H1229 cells treated with NSCLC-Exo exhibited enhanced proliferation capacity (Figure 2(a)) and colony-forming ability (Figure 2(b)), while cell apoptosis was significantly suppressed (Figure 2(c)). In addition, cell migration (Figure 2(d)) and invasion (Figure 2(e)) were also significantly augmented in NSCLC-Exo group. We also qualified the relative level of circPLK1 level in NSCLC-exosome and control exosome by qRT-PCR. NSCLC-derived serum exosomes showed a significantly higher level of circPLK1 than exosomes from healthy controls (Figure 3(f)). These results suggest that NSCLC-derived serum exosomes promote the malignant transformation in NSCLC cells.

### Overexpression of circPLK1 promotes proliferation, migration, and invasion of NSCLC cells

To directly investigate the functional role of circPLK1 in NSCLC cells, A549 and H1229 cells stably overexpressing circPLK1 were established (see methods). qRT-PCR analysis confirmed that in the circPLK1 overexpressing cells, circPLK1 expression increased by 10 folds as compared to the cells expressing empty vector (Figure 3(a)). We then performed functional assays to examine the malignant phenotype in NSCLC cells with circPLK1 overexpression. A549 and H1229 cells treated with circPLK1 overexpression exhibited enhanced proliferation capacity (Figure 3(b)) as well as augmented colony-forming ability (Figure 3(b)), while cell apoptosis was significantly suppressed by circPLK1 overexpression (Figure 3(d)). The cell migration (Figure 3(e)) and invasion (Figure 3(f)) were also significantly augmented in circPLK1 overexpression group. These results indicate that circPLK1 overexpression promotes the malignant phenotype in NSCLC cells.

### Identification of circRNA–miRNA–mRNA regulatory network

**Figure 4.**
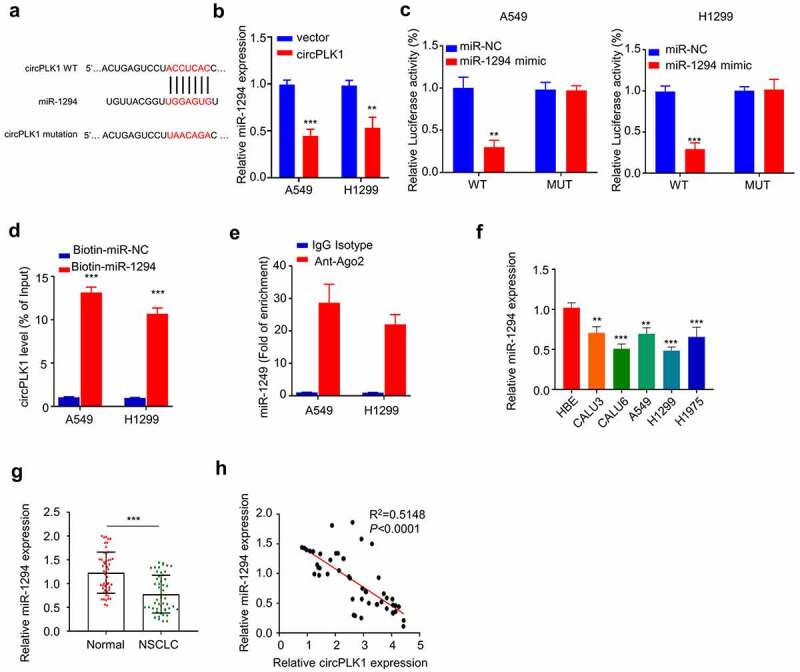
CircPLK1 targets miR-1294. (a) Starbase predicted that CircPLK1 targeted miR-1294. (b) The expression level of miR-1294 was detected by qRT-PCR in circPLK1 overexpression cells. (c) Dual luciferase reporter assay using WT and mutated reporter. (d) The interaction between circPLK1 and miR-1294RNA pull-down assay. (e) Anti-Ago2 RIP assay, IgG isotype was used as negative control. (f) The expression level of miR-1294 in NSCLC cell lines was detected by qRT-PCR. (g) The expression level of miR-1294 in NSCLC and adjacent normal tissues was detected by qRT-PCR. (h) Spearman correlation coefficient was used to analyze the correlation between circPLK1 and miR-1294 expression in 50 NSCLC tissues. *P < 0.05; **P < 0.01; ***P < 0.001.

To identify the potential target miRNAs of circPLK1, we searched the non-coding RNA databases StarBase v3.0 and found that miR-1294, a tumor suppressor in various tumors, contains a putative motif for circPLK1 binding (Figure 4(a)). A549 and H1229 cells overexpressing circPLK1 showed a significantly lower level of miR-1294 expression (Figure 2(b)). To validate their functional interaction, we transfected A549 and H1229 cells with luciferase reporter containing WT binding site or the mutated reporter (mut) in the presence of miR-NC or miR-1249 mimic. The luciferase activity of WT reporter was significantly suppressed by miR-1249 mimic, while no effect was observed for mutated reporter (Figure 4(c)). To directly show their interaction, we transfected A549 and H1229 cells with biotin-miR-1294 probe, with the biotin-miR-NC as the negative control probe. Streptavidin-biotin-based RNA pull-down assay showed that biotin-miR-1294 probe could precipitate circPLK1 to a much higher degree when compared to the nonspecific miR-NC probe (Figure 4(d)). In the meanwhile, anti-Ago2-based RNA-immunoprecipitation assay further demonstrated that anti-Ago2 antibody could significantly enrich miR-1249, when compared to the IgG isotype control (Figure 4(e)). Together, these data strongly indicate circPLK1 interacts with miR-1249.

Additionally, we determined the expression levels of miR-1294 in NSCLC cell lines (CALU3, CALU6, A549, H1229, and H1975) and HBE cells. The results showed that miR-1294 expression was significantly decreased in NSCLC cells compared to HBE cells (Figure 4(f)). miR-1294 expression was also significantly reduced in NSCLC tissues as compared to the adjacent normal tissues (Figure 4(g)). In NSCLC tissues, there was a significant negative correlation between the expression levels of circPLK1 and miR-1294 (Figure 4(h)). Our findings collectively suggest that miR-1294 is target of circPLK1 and is negatively regulated by circPLK1 in NSCLC cells.

**Figure 5.**
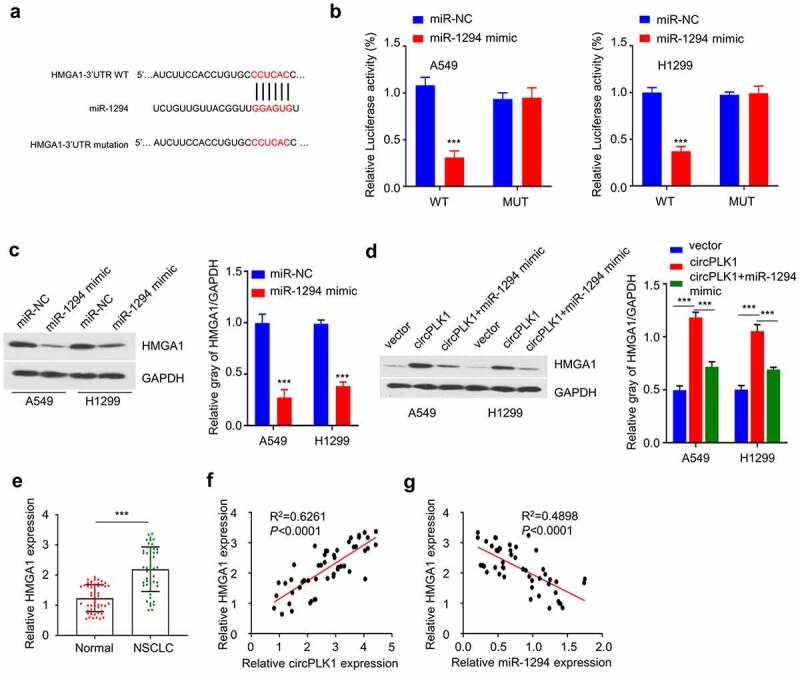
CrcPLK1 elevates the expression of HMGA1 via sponging miR-1294. (a) The presence of miR-1294 binding sites in the 3 ‘non-coding region of HMGA1 mRNA, as predicted by STARBASE. (b) Dual Luciferase reporter assay was performed in A549 and H1229 cells, using WT and mutated reporter. (c) The protein level of HMGA1 in A549 and H1229 cells transfected with miR-1294 mimic was detected by Western blot. (d) The protein level of HMGA1 in different groups of A549 and H1229 cells was detected by Western blot. (e) The expression level of HMGA1 in NSCLC and adjacent tissues was detected by qRT-PCR. (f) Spearman correlation coefficient was used to analyze the correlation between HMGA1 and miR-1294 expression in 50 NSCLC tissues. (g) Spearman correlation coefficient was used to analyze the correlation between HMGA1 and circPLK1 expression in 50 NSCLC tissues. *P < 0.05; **P < 0.01; ***P < 0.001.

To fully understand the downstream target of miR-1294, we searched StarBase v3.0 for the target mRNA of miR-1294, which identfied the complementary sequence between the 3ʹUTRof HMGA1 mRNA and miR-1294 (Figure 5(a)). Dual-luciferase reporter assay demonstrated that the luciferase activity of WT reporter was significantly suppressed by miR-1249 mimic, while no effect was observed for mutated reporter (Figure 5(b)). In the meanwhile, miR-1249 mimic transfection significantly reduced the protein level of HMGA1 (Figure 5(c)). We also applied miR-1249 mimic in A549 and H1229 cells overexpressing circPLK1, which showed that circPLK1 overexpression upregulated HMGA1 and this effect was largely abrogated by miR-1249 mimic (Figure 5(d)). Together, these data suggest that HMGA1 is target mRNA of miR-1294 and circPLK1 regulates HMGA1 expression via targeting miR-1294. This observation was further supported by the upregulated level of HMGA1 expression in NSCLC tissues when compared to adjacent normal tissues (Figure 5(e)). Additionally, Spearman’s rank correlation analysis showed that in NSCLC tissues there was a positive correlation between with circPLK1 and HMGA1 expression (Figure 5(f)), and a negative correlation between miR-1294 and HMGA1 expression (Figure 5(g)). In summary, these results collectively show that circPLK1 promotes HMGA1 expression in NSCLC cells by sponging miR-1294.

### circPLK1 promotes malignant phenotype of NSCLC cells through miR-1229/HMGA1 axis

**Figure 6.**
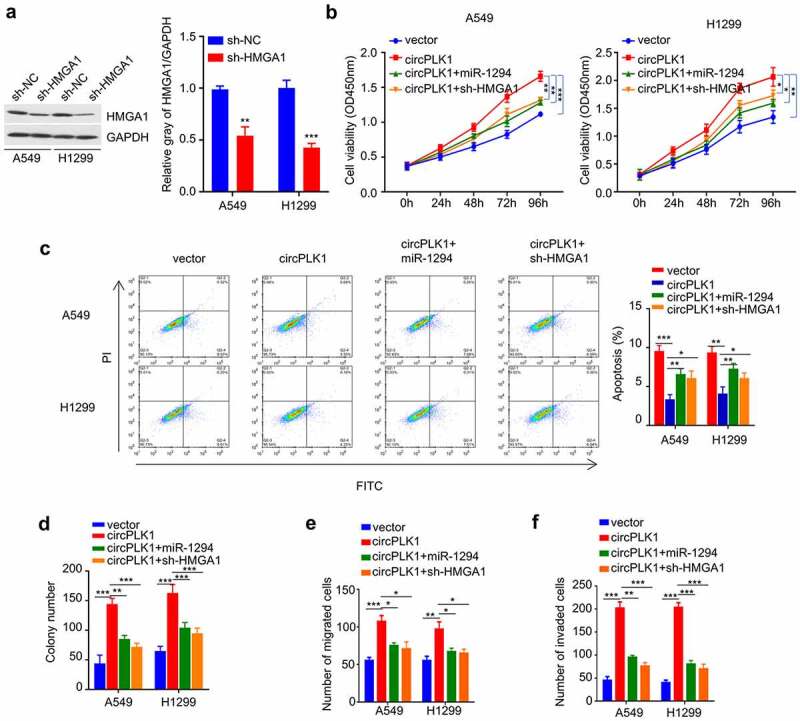
CircPLK1 regulates NSCLC through the miR-1294/HMGA1 axis. (a) The protein level of HMGA1 after transfecting with shRNA against HMGA1 was detected by Western blot. (b) CCK-8 assay was used to detect cell proliferation. (c) Colony formation assay was used to examine the ability of colony formation. (d) Apoptosis was detected by flow cytometry. (e) Transwell assay to measure migration ability. (f) Transwell assay to measure invasion ability. *P < 0.05; **P < 0.01; ***P < 0.001.

We further evaluated the functional involvement of circPLK1/miR-1229/HMGA1 axis in regulating the malignant phenotype of NSCLC cells. We first established the knockdown of HMGA1 by transfecting sh-HMGA1 into A549 and H1229 cells. As shown in Figure 6(a), the level of HMGA1 protein was notably reduced after HMGA1 knockdown. We then used the established A549 and H1229 cells with circPLK1 overexpression, and transfected the circPLK1-overexpressing cells with miR-1229 mimic or sh-HMGA1. We then performed functional assays to determine whether miR-1229 mimic and HMGA1 silencing affect the functional role of circPLK1. We observed that the enhanced cell proliferation by circPLK1 overexpression was partially suppressed after co-transfecting miR-1294 mimic or HMGA1 knockdown (Figure 6(b)). Similar effects were observed in colony-formation assay (Figure 6(c)). miR-1294 mimic transfection or HMGA1 knockdown could also increase the percentage of apoptotic cells in circPLK1-overexpressing cells (Figure 6(d)). In the meanwhile, transwell migration and invasion assay showed that the augmented cell migration and invasion due to circPLK1-overexpression were attenuated by miR-1294 mimic or HMGA1 knockdown (Figure 6(e,f)). These results suggest that miR-1294/HMGA1 axis mediates the functional role of circPLK1-overexpression in NSCLC cells.

### NSCLC-exosome promotes malignant phenotype of NSCLC cells through miR-1229/HMGA1 axis

**Figure 7.**
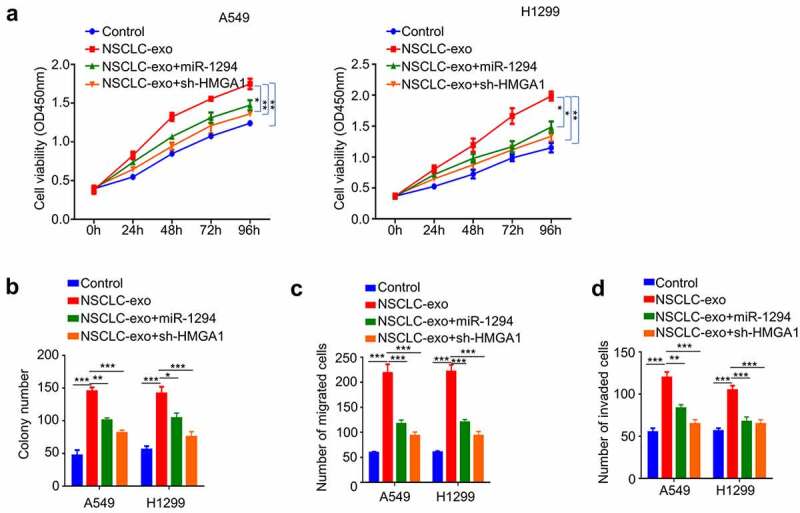
NSCLC-exosomes promotes the malignant phenotype of NSCLC through the miR-1294/HMGA1 axis. Cells treated with NSCLC-exosomes were transfected with miR-1294 mimic or shRNA targeting HMGA1. (a) CCK-8 assay was used to detect cell proliferation. (b) Colony formation assay was used to examine the ability of colony formation. (c) Transwell assay to measure migration ability. (d) Transwell assay to measure invasion ability. *P < 0.05; **P < 0.01; ***P < 0.001.

Since NSCLC-exosome contains a high level of circPLK1 (Figure 2(f)), and also promotes the malignant phenotype of NSCLC cells, we next attempted to validate whether miR-1229/HMGA1 axis also account for the effect of SCLC-exosome. A549 and H1229 cells treated with NSCLC-exosome were transfected with miR-1229 mimic or sh-HMGA1. We observed that the enhanced cell proliferation by NSCLC-exosome was partially suppressed after the transfection of miR-1294 mimic or HMGA1 knockdown (Figure 7(a)). Similar effects were observed in colony-formation assay (Figure 7(b)). In the meanwhile, the augmented cell migration and invasion ability by NSCLC-exosome was also attenuated by miR-1294 mimic or HMGA1 knockdown (Figure 7(c,d)). Overall, our data support the idea that the malignant phenotype enhanced by exosome-derived circPLK1 is mediated by miR-1294/HMGA1 axis in NSCLC cells.

## Discussion

Exosomes are extracellular vesicles implicated in various physiological and pathological processes [15,16]. With the development of high-throughput proteomics and genomics, exosomes are found to contain diverse functional molecules that could mediate short-distance and long-distance cellular communication, allowing the exchange of proteins, lipids, and genetic materials. Exosome-mediated cellular is a highly conserved communication that is vital for both normal cells and tumor cells [17,18]. In recent years, exosomes have been proposed as disease biomarkers and prognostic factors, with clinical applications in diagnosis and therapy [17–20]. Besides, exosomes have the potential to serve as carriers for gene and drug delivery [21–24]. For example, two studies demonstrated that the early diagnosis rates of lung cancer and pancreatic cancer could reach 75% and 90%, by analyzing the exosomes in patients’ blood [25,26]. Tumor cell-derived exosome-specific proteins or small molecule RNA (miRNA) can be used for tumor diagnosis and as indicators for tumor invasion [27–29].

Circular RNAs are non-coding RNAs with closed and circular structures [30,31]. They are highly abundant and are expressed in a tissue-specific manner. CircRNAs regulate gene expression at transcriptional and post-transcriptional stages, rendering them excellent candidates as clinical diagnostic markers or therapeutic targets in humans [32,33]. In particular, the abundance and stability of circRNAs in exosomes have been confirmed by previous studies, which may favor the formation of pre-metastatic foci in tumor microenvironment [34–37]. In our study, we found that circPLK1 is highly abundant in the exosomes derived from the serum of NSCLC patients, and NSCLC-exosomes could promote the malignant phenotype pf NSCLC cells. These data suggest that NSCLC-exosomes may function to promote NSCLC progression through the activity of circPLK1. However, whether the exosomes originate from cancer cells or the cells in the tumor microenvironment need to be further determined.

Exosome-derived non-coding RNAs are widely reported in tumor biology. For example, exosome-derived circRASSF2 were shown to facilitate the progression of laryngeal squamous cell carcinoma (LSCC) [38]. Compared with the control group, the expression level of circRASSF2 in LSCC tumor tissue was significantly increased [38]. Additionally, interfering the activity of circRASSF2 through the miR-302b-3p/IGF-1 R axis could greatly suppress cell proliferation and migration [38]. Meanwhile, an increasing body of evidence indicates that the interactions between cancer cells and the surrounding matrix promote metastasis. Tumor-released exosomal circRNA PDE8A promotes invasive growth via the miR-338/MACC1/MET pathway in pancreatic cancer, which may be involved in the remodeling of the extracellular matrix [39]. CircPDE8A secreted by tumors may enter the bloodstream through exosomal transport, where it acts as a sponge for miR-338, which in turn regulates MACC/MET/ERK and AKT pathway to promote invasive metastasis [39]. Similarly, a recent study showed that the exosomal circNRIP1 sponges miR-149-5p to promote gastric cancer proliferation and migration [40]. In our study, we showed that exosome-derived circPLK1 also enhanced the migration and invasion of NSCLC cells by targeting miR-1294/HMGA1 axis. It remains to be determined whether circPLK1/miR-1294/HMGA1 axis also modulate the extracellular matrix remodeling.

PLK1 (polo-like kinase 1) is a member of PLK family kinases, which is essential for the mitosis [41]. PLK1 mutation is strongly associated with the emergence and progression of malignant tumors and is considered as one of the most promising targets for targeted anti-cancer therapy [41,42]. The circular RNAs derived from PLK1 locus also have important roles in tumor biology. A previous study showed that silencing circPLK1 could inhibit the growth of breast cancer tumors by regulating miR-4500/IGF1 axis [10]. Additionally, circPLK1 can promote the malignant phenotype of triple-negative breast cancer by adsorbing tumor suppressor miRNA [43]. In our study, we further show that the exosomal circPLK1 in NSCLC facilitates the malignant progression of NSCLC cell through the circPLK1/miR-1229/HMGA1 axis. However, the mechanisms by which circPLK1 is upregulated and secreted in exosomes in NSCLC remain to be elucidated.

HMGA1 has been recognized as an oncogene, which is frequently overexpressed and implicated in tumor initiation and progression [44]. Overexpression of HMGA1 has been associated with the advanced grading, recurrence and poor prognosis of bladder cancer [45,46]. In triple-negative breast cancer, HMGA1 overexpression can promote tumor invasion and metastasis [47]. In a colorectal cancer study, researchers showed that HMGA1 could be targeted by miR-26a to modulate the expression [48,49]. Our study identified HMGA1 as a miR-1229 target in NSCLC, which serves as a downstream effector to mediate the functional role of circPLK1. These findings support the notion that elevated HMGA1 expression is an oncogenic factor to promote cancer progression.

## Conclusion

In summary, we demonstrated that circPLK1 is upregulated in serum exosomes from NSCLC patients, NSCLC tissues, and NSCLC cells. CircPLK1 overexpression could increase HMGA1 level by sponging a miR-1294, thus promoting the malignant progression of NSCLC. Our study suggests that circPLK1 derived from serum exosomes might serve as a potential prognostic marker for NSCLC.

## Highlights

- CircPLK1 was upregulated in serum exosomes and tissues from NSCLC patients.
- NSCLS-exosomes and circPLK1 promote malignant phenotype in NSCLC cells.
- CircPLK1 regulates NSCLC progression by targeting miR-1294/HMGA1 axis.
